# An Analysis of Vocal Features for Parkinson’s Disease Classification Using Evolutionary Algorithms

**DOI:** 10.3390/diagnostics12081980

**Published:** 2022-08-16

**Authors:** Son V. T. Dao, Zhiqiu Yu, Ly V. Tran, Phuc N. K. Phan, Tri T. M. Huynh, Tuan M. Le

**Affiliations:** 1 School of Industrial Engineering and Management, International University, Vietnam National University, Ho Chi Minh City 700000, Vietnam; 2 Department of Industrial Management, National Taiwan University of Science and Technology, Taipei City 106, Taiwan; 3 School of Electrical Engineering, International University, Vietnam National University, Ho Chi Minh City 700000, Vietnam

**Keywords:** Parkinson’s disease, grey wolf optimization, feature subset selection

## Abstract

Parkinson’s Disease (PD) is a brain disorder that causes uncontrollable movements. According to estimation, roughly ten million individuals worldwide have had or are developing PD. This disorder can have severe consequences that affect the patient’s daily life. Therefore, several previous works have worked on PD detection. Automatic Parkinson’s Disease detection in voice recordings can be an innovation compared to other costly methods of ruling out examinations since the nature of this disease is unpredictable and non-curable. Analyzing the collected vocal records will detect essential patterns, and timely recommendations on appropriate treatments will be extremely helpful. This research proposed a machine learning-based approach for classifying healthy people from people with the disease utilizing Grey Wolf Optimization (GWO) for feature selection, along with Light Gradient Boosted Machine (LGBM) to optimize the model performance. The proposed method shows highly competitive results and has the ability to be developed further and implemented in a real-world setting.

## 1. Introduction

Parkinson’s disease is a slowly progressive degenerative disorder characterized by tremors, increased muscle tone, decreased locomotor and bradykinesia, and eventually stabilization of posture and/or gait. The condition is a progressive non-curable disorder that affects movements neurologically. Tremor, fatigue, postural disturbance, anxiety, dysphonia, pain, and cognitive problems are some of the common symptoms of PD [1,2]. Over ten million people around the world undergo PD. Necrosis in different brain regions can lead to PD, which is a common degenerative motor system disorder. People with parkinson (PWP) undergo many severe symptoms that negatively impact their ability to function correctly and endanger their safety. Healthcare services for PD patients remotely through the use of information technology systems rely on assessing behaviours intensity using indirect devices. Dysphonia is the common symptoms that appear in the early stages of this disease.

One of the previous diagnostic criteria for Parkinson’s disease, the UK Parkinson’s Disease Society Brain Bank Clinical Diagnostic Criteria, also known as Queen Square Brain Bank criteria, was the most used [3]. According to this criterion, the diagnosis of Parkinson’s disease consists of three steps. The first step is determine the presence of Parkinson’s syndrome with decreased or slow movement (bradykinesia) associated with one or three symptoms of 4–6 Hz rest tremor, rigidity, and postural instability. The second step is tidentify any exclusion criteria in the history or physical examination. The third step is to have at least three supporting criteria for a definitive diagnosis of Parkinson’s disease. However, the pathophysiology of Parkinson’s disease is increasing, these clinical diagnostic criteria are not sufficient to increase the diagnostic accuracy of the disease. The International Parkinson and Movement Disorder Society has developed the MDS (Movement Disorder Society Clinical Diagnostic Criteria for PD) criteria for the clinical diagnosis of Parkinson’s disease. Compared with the previous diagnostic criteria of the British Parkinson’s Disease Association, the clinical diagnosis of Parkinson’s syndrome of MDS requires only bradykinesia accompanied by at least one of the following two criteria, including: rest tremor and rigidity. The postural disequilibrium criterion was omitted because this symptom only appeared when the disease had progressed for a long time and was not helpful in the early diagnosis of Parkinson’s disease [4,5].

By adequately examining speech signals, neurophysiologists can analyze non-motor symptoms, thus providing timely patient predictions. Research papers for PD are proposed to give timely and advanced recognition by using Machine Learning (ML) based on vocal signals, along with many researchers’ works to enhance prediction accuracy [6,7]. Machine Learning (ML) includes many different algorithm-based methods that study the dataset by analyzing and detecting patterns to further enhance predicting the outcomes. ML has contributed to the field of healthcare by detecting multiple diseases and enabling specialists to achieve great results. Supervised learning is commonly used in classification problems by training data using machine algorithms with the corresponding outputs [8,9,10,11]. Research papers for PD are proposed to give timely and advanced recognition by using Machine Learning (ML) based on vocal signals, along with many researchers’ works to enhance prediction accuracy.

Due to the similarity in symptoms, biopsy or diagnostic tests are implemented to rule out the possibility of other diseases rather than detecting PD (Cova & Priori, 2018) [2]. These methods of examination are not only costly, but they are also prone to false results. Yunusova et al. (2008) [12] stated that even in the early stages, PD can vocally affect patients; hence, vocal features have been successfully implemented to predict and monitor PD. Many researchers have applied machine learning approaches to obtain high accuracy results. Machine Learning is extensively being implemented across many research fields, including neurology, to examine PD. By utilizing Machine Learning, we can significantly reduce the workload of medical staff and the inconvenience of direct clinical examinations, most importantly, improve diagnosis efficiency.

Morphological or lexical vocal features cannot be fully captured by sustained phonation of vowels. However, (Gürüler, 2017) demonstrates that they are adequate for differentiating PWP from healthy individuals. All data collected to do this research is assumed to be accurate at the time of collecting from the Department of Neurology, Cerrahpaşa Faculty of Medicine, Istanbul University, Turkey. This public dataset is retrieved from UCI Machine Learning Repository and consists of 252 subjects.

This study proposes an approach based on Machine Learning to classify speech signals into healthy people and people affected by Parkinson’s disease (PWP). The proposed method will be applied to the public PD classification dataset retrieved from UCI Machine Learning Repository. This research aims to improve classification performance by maximizing the accuracy of the machine learning algorithm.

## 2. Literature Review

### 2.1. Related Works

Based on speech signals, numerous studies on the classification of PD have been researched, applying different feature engineering and classifying methods to analyze voice records. The techniques proposed have achieved satisfactory results with high accuracies for the same dataset used in this research.

Besides baseline features used in previous studies, Sakar et al. [13], also extracted MFCC (Mel-Frequency Cepstral Coefficients), WT (wavelet transform), and TQWT (Tunable Q-factor Wavelet Transform)—based features for classifying PWP (people with parkinson). The results of this investigation showed that TQWT features outperformed the modern voice features widely employed in PD classification. When mRMR feature subset selection was applied to the SVM-RBF (Support Vector Machine—Radial Basic Function) classifier, the combination of TQWT and MFCC features improved the performance, resulting in an accuracy of 86 percent and an F-measure of 0.84.

With the use of TQWT-based features and this size-improved dataset implemented by Sakar et al. [13]. The features extracted by single value decomposition continued to be selected using the relief-based method to obtain the 50 most significant features in Tuncer et al. [14]. Afterwards, K-NN (K-Nearest Neighbor), the simplest benchmark classifier, is utilized to achieve high classification performance even with a heterogeneous dataset. In the implementation, k is set to 1 for the best performance after testing several k values, and city block (Manhattan) is used as the distance metric.

Polat [15] presented an approach employing a combination of a Random Forest (RF) and the Synthetic Minority Over-Sampling Technique (SMOTE). Without using SMOTE, researchers get an accuracy of 87.037% and 94.89% by oversampling the minority class with RF classifier. Over-sampling can balance the classes, but it can also increase the likelihood of overfitting since it replicates the oversampled class data points.

Solana-Lavalle & Rosas-Romero [16] proposed a combination of Wrapper Feature Selection and SVM classifier to obtain 94.7% accuracy on the larger scale of the dataset. The feature subset selection method used in this research did not account for the biological and vocal features and instead selected the best K features to feed into the machine learning model.

A small and limited number of features are selected from a total of 754. This leads to the loss of important information, which from TQWT and WT based features, since it is an extensive ways to quantify frequency deviations in signals of speech and comprise at least ten original features each. 

Other Deep Learning algorithms are utilized in detecting Parkinson disease; for instance, Gunduz [17] introduced frameworks using Convolutional Neural Networks (CNN). Firstly, a combination of all features into 9—layer CNN, while the other framework inserted the feature sets to parallel input layers linked to the convolution layers. By employing TQWT and baseline features, they achieved an accuracy of 84.9%. Employing triple feature sets that applied WT, TQWT, and baseline characteristics, this was increased to 86.9%. 

### 2.2. Feature Selection

**Figure 1:**
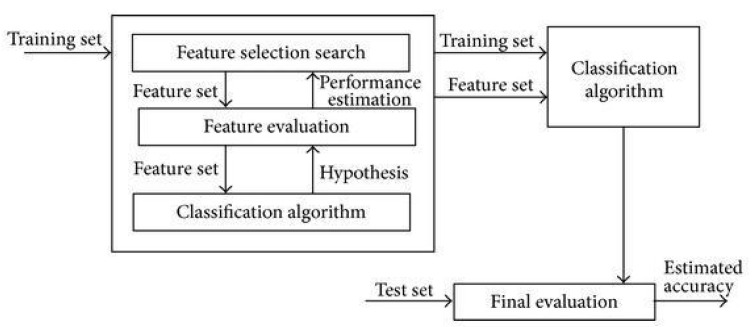
Flowchart of wrapper-based approach.

The goal of the feature selection method is to choose a few important features from a large dataset. Generally, feature subset selection methods can be divided into two methods: wrapper and filter. The filter method is not dependent on the machine learning algorithm. It uses mutual and statistical information to search the features (Wang et al., 2014 [18]). On the other hand, the wrapper method used a specific classifier to evaluate the subset of features. The wrapper method can contribute to better performance and is widely applied in several feature selection problems. The following Figure 1 indicates the flowcharts of the wrapper method.

Nevertheless, a greater number of computations of the wrapper method is required to get the final feature subset than the filter method. Hence, wrapper models are more computationally costly than filter models. In addition, the wrapper method often causes overfitting since it has the classification algorithm in the process; therefore, the selected subset of features is unavoidably biased to the predefined classifier. It is recommended that testing (hold-out) data be used after selecting the final subset to tackle the problem.

For the Feature Selection process, an Enhanced Binary Grey Wolf Optimization (EBGWO), inspired by (Kohli et al., 2019 [19]) study, has been chosen to be the search algorithm and an induction classifier with low complexity is preferable to ensure the goodness of the selected features. The K-NN method is simple and a common machine learning classifier. In this system, K-NN is utilized as the induction classifier.

### 2.3. Classification Algorithms

#### 2.3.1. Support Vector Machine (SVM)

SVM algorithms are both effective and flexible for the problem of classification as well as regression. It illustrates multiple classes in a hyperplane in multi-dimensional space. The hyperplane will be constructed iteratively by SVM in order to minimize error. Support vectors are the closest data points to the hyperplane that manipulate the hyperplane position and orientation. SVM aims to segregate the classes to find a maximum marginal hyperplane by using support vectors and maximizing the range of the classifier.

#### 2.3.2. Nearest Neighbor (KNN)

K-NN is based on the hypothesis that cases in a dataset can be found adjacent to other instances with similar properties. If the instances are marked with a classification label, then an unclassified instance’s label value can be obtained by evaluating the class of its nearest neighbours. The k-NN locates the k nearest instances to the query instance and determines its class by defining the single most frequent class label. K-NN is a simple algorithm to interpret and useful for both regression and classification problems, and there is no need to make additional assumptions, tune several parameters, or build a model.

#### 2.3.3. Decision Tree (DT)

Decision Tree is simple to understand and interpret. It requires minimal data preparation compared to other algorithms. DT handle both numerical and categorical data as well as multiple output problems. Using boolean logic to explain the situation where it can be seen in a model is known as the white-box model. This also provides the ability to use statistical tests to validate the reliability of a model. Even when the real model from which the data were derived slightly violates DT’s assumptions, it still performs well.

#### 2.3.4. LightGBM (LGBM)

LGBM was created and introduced to the world by Microsoft in 2016. It can be applied in regression and classification problems. LGBM is a gradient boosting framework developed that relies on the basis of DT in a serial manner. Ma et al. [20] (2018) concluded that the algorithm is capable of achieving higher accuracy, good precision, low memory consumption, and breakneck training speed even when working with an extremely large dataset of high dimensionality. However, this algorithm is very sensitive to overfitting when applied to a small dataset (Ke et al., 2017 [21]). LGBM differentiates itself from other boosting algorithms by extending leaf-wise instead of level-wise. The splitting occurs at the leaf with the highest loss to decline more loss than a level-wise method.

## 3. Methodology

**Figure 2:**
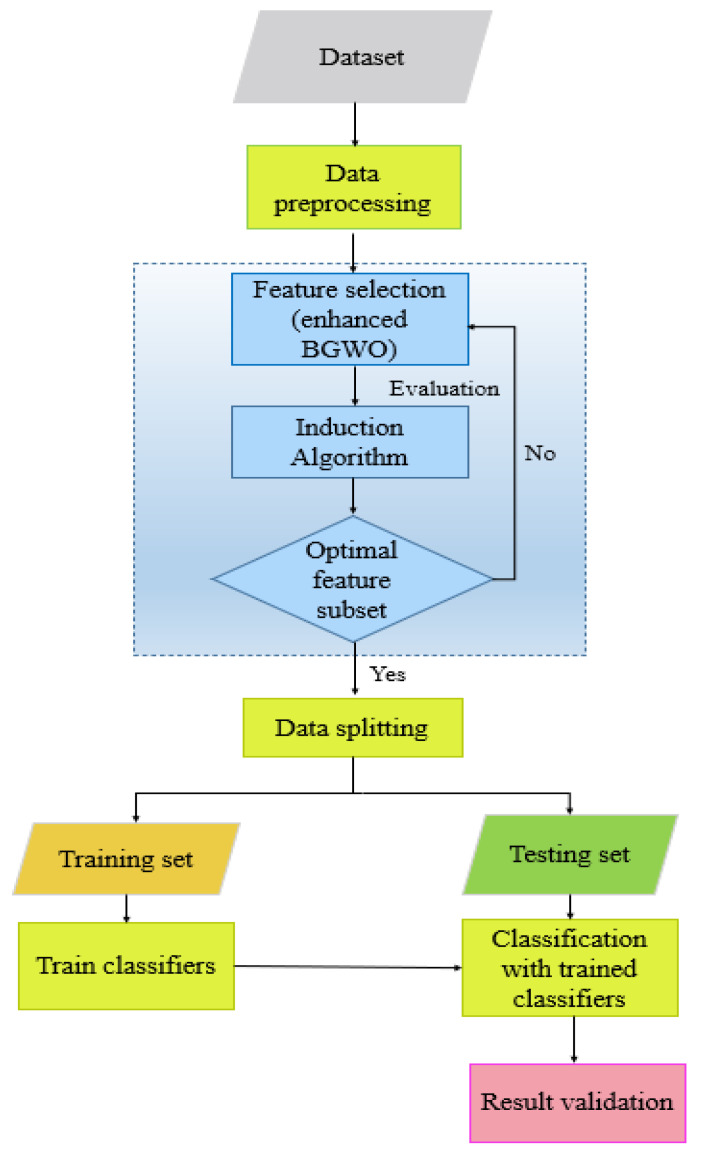
Proposed model applying machine learning algorithms.

In this research, the dataset is downloaded directly from the public source UCI Machine Learning Repository that Sakar et al. [13] provided at the Department of Neurology in Cerrahpaşa Faculty of Medicine, Istanbul University. Data standardization is in charge of converting data into a common scale without distorting differences in the ranges of values. The data cleaning process will be implemented to test for missing or noisy values, and then we will proceed to remove any incomplete entries. Then, the features are selected and extracted respectively to maintain the relevant feature subsets assisting the classification performance. In addition, this metaheuristic requires a small number of parameters, which is easier to implement and makes it superior to earlier ones. Through the interaction of individuals in the population, the GWO algorithm seeks for the optimal regions in the complex search space. After that, we used these selected features to enhance the architecture of four machine learning models. The proposed methodology is presented in this section. The process of our system is depicted in Figure 2.

### 3.1. Parkinson’s Disease Dataset

This research uses the publicly available dataset provided by Sakar et al. [13] through their own study. The dataset was gathered from a total of 252 individuals, including 188 PWP and 64 healthy individuals. There are 107 males and 81 females within the PWP group with the age range of 33 to 87 (65.1 ± 10.9), followed by the healthy group, which consists of 23 males and 41 females, with a relatively close range from −41 to 82 (61.1 ± 8.9). It is required that the preceptor device is tuned to 44.1 kHz to record the vowel /a/ and repeat it three times for each subject.

Jitter and shimmer variances, fundamental frequency parameters, harmonicity parameters, Recurrence Time Density Entropy (RPDE), Detrended Fluctuation Analysis (DFA), and Pitch Period Entropy (PPE) are used vocal features in many researches [13,22,23], also referred to those as baseline features. Other features are also extracted such as formant frequencies, intensity parameters, and bandwidth utilizing the software that Boersma, P., (2006) developed for acoustic analysis.

Automatic speech recognition, Speaker recognition [24], biomedical voice recognition [25], and PD diagnosis [26] have all used Mel-Frequency Cepstral Coefficients (MFCCs) as a robust technique for extracting features from voice signals. To integrate cepstral analysis and spectral-domain partitioning, MFCCs employ triangular overlapping filter banks. This feature set is also utilized in PD research to timely detect changes in articulatory movements [26]. In the current researched data, the 84 features related to MFCCs are based on the mean and standard deviation which is calculated from the original 13 MFCCs, in addition to their first and second derivatives [13] and the log-energy of the signal.

The determination regarding signals, especially when there are minor fluctuations on a regional scale, the wavelet transform (WT) is a popular method. Several researchers have used WTs extracted features from the fundamental frequency (F0) to predict PD. WT-based features are used to record the degree of deviation in voice samples [27]. Therefore, in diseased speech samples, rapid alterations in the complete periodicity of long-term vowels would be recognized. In order to extract WT-based features from F0 and the log transformation during data collection, voice signals are subjected to a 10-level discrete wavelet transformation.

The Shannon’s energy, log energy entropy, and Teager-Kaiser energy of both detailed coefficients and approximation are among the 182 WT-based attributes produced by this procedure.

Tunable Q-factor wavelet transform (TQWT) is another robust feature extraction method that transforms signals in a better quality according to their behaviour using three tunable parameters: redundancy (r), Q-factor (Q), and a number of levels (J).

The time-domain properties of the speech signals are taken into account while setting the parameters of the TQWT in the dataset was utilized. This occurs in the order of the value of the Q-factor parameter, which is specified to govern the oscillatory behavior of wavelets. The value of a parameter must therefore be adjusted to equal or more than three to prevent the undesirable ringing in wavelets. Several levels (J) are searched for in the specified intervals to determine the accuracy values of the different Q-r pairs. In this dataset, some experiments result in 432 TQWT-related features [13]. An overview of some of the features set are depicted in Table 1 below.

### 3.2. Preprocessing Data

**Figure 3:**
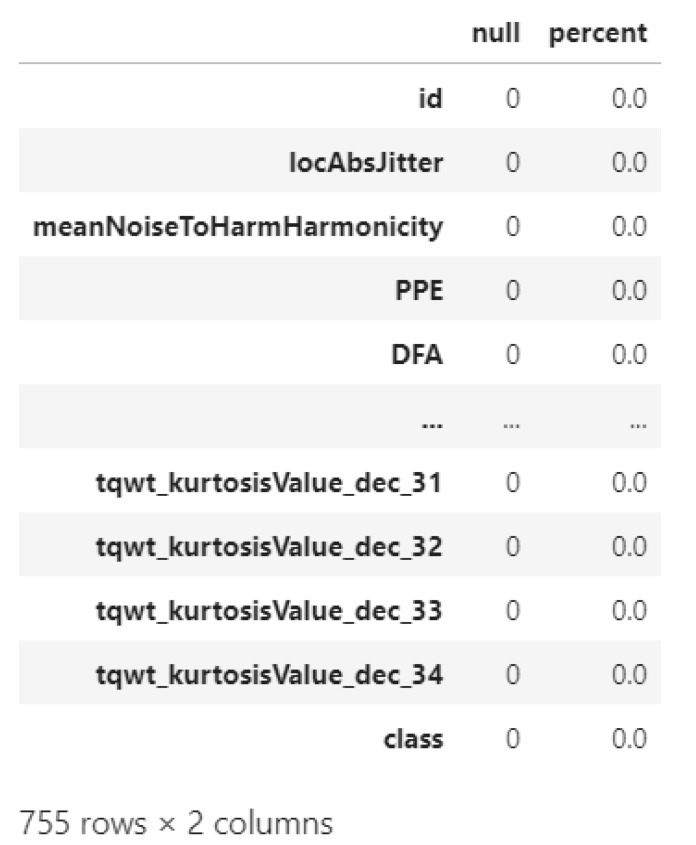
Checking for a missing value.

The data comprises 755 features (columns) and one label (output feature) whose value is binary, which determines whether the patient’s health condition will fall into having PD or not. Afterwards, missing values must be detected by using the IsNull() function, and their row will be erased. As shown in the Figure 3, there is no missing value; hence, no adjustment is needed.

Data standardization is the method of transforming data into a standard format so that users can evaluate and process it. Data standardization is crucial for many reasons. Different columns of data with a different range of values, and some will have negative values while some will have positive ones; some will be integer whereas some will be decimal. If the data are not converted into one standard format, it is difficult for them to be compared and analyzed. Therefore, standardization is a rescaling technique to make the mean of the variable’s values should be centered around 0 and the standard deviation around 1.

**Figure 4:**
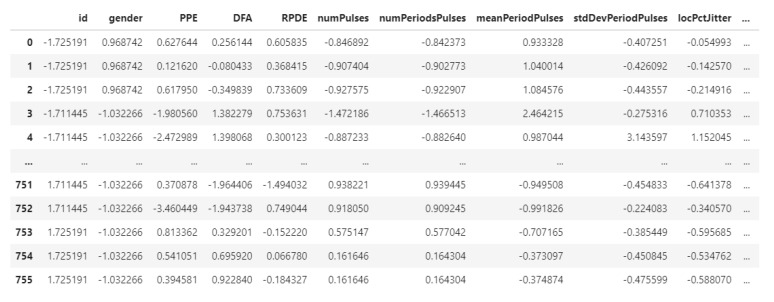
The output of data standardization.

Data standardization is sometimes called Z-score normalization. The Z-score, known as the benchmark score of the model, is the transformed value for each data point. To normalize the dataset using standardization, we take every x value inside the data set and transform it into the corresponding z value using the following formula:

(1) $$x^{\prime }=\frac{x-\mu }{\sigma },$$

where $x^{\prime }$ is the normalized value of each feature, *x* is the original value, *μ* is the mean, and *σ* is the standard deviation of the feature values. From the dataset, the first and last five rows are depicted in Figure 4.

**Figure 5:**
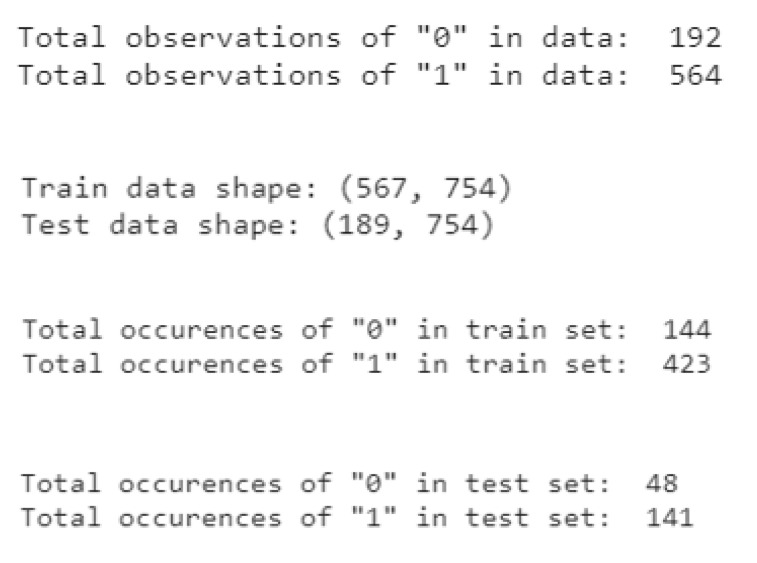
The output of data splitting.

The feature data shape (756, 754) is split into train data and test data, with a ratio of 75/25. Hence, the data size of the train set is (567, 754), and that of the test set size is (189, 754) which are illustrated in Figure 5. Additionally, the feature data is split in a stratified manner, which reserves the proportion of labels 0 and 1 from the feature data in both train and test sets:

### 3.3. Grey Wolf Optimization

Grey Wolf Optimization (GWO) [28] is a popular metaheuristic algorithm inspired by the behaviour of wolves in their natural habitat, specifically in their ways of hunting prey. GWO has been demonstrated to converge to the optimal solution and is a computationally viable method. The essential idea of GWO is that knowledge is enhanced not only through personal experience but also via social interaction among the population’s candidates.

**Figure 6:**
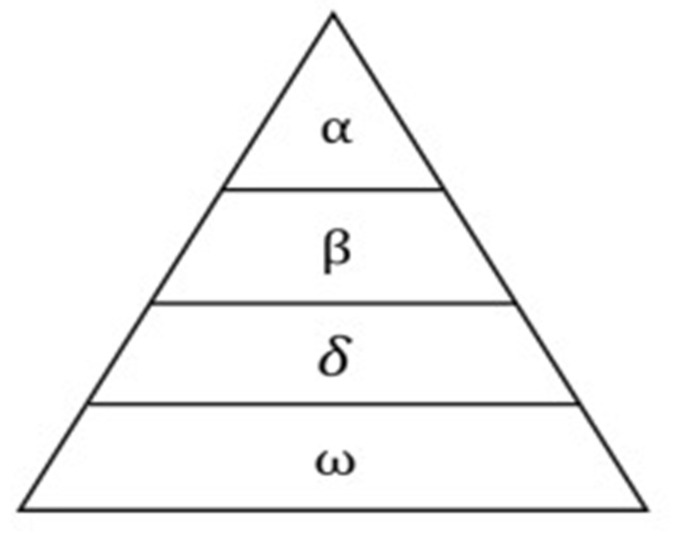
Hierarchy of wolves in GWO.

Grey wolves are likely to go hunting in a group or a pack. The organization system and the behaviour of wolves are clearly described in a study by Mirjalili (2014). Wolves have a strict social hierarchy to be followed, which is a top-down dominance. The highest level of wolves is called alpha, which can be either male or female, whose responsibility is to manage and lead the pack in hunting, moving, sleeping time, and so on. Alpha maybe not be the strongest in physical health but be the strongest in intellectual problem-solving. The other wolves usually show their respect and obedience by pulling their tails on the ground. The second-highest level is beta, which is the direct subordinate of alpha. Beta makes other lower-level wolves obey and follow the decision of alpha. Moreover, it helps alpha in decision-making problems and will be the next candidate for the alpha wolf position if the current alpha wolf is passed away. The lowest level is omega wolves, who have no subordinates but are the subordinates of all other wolves. Omega always must submit and report to other wolves in the organization. As they are listed as the last ranked in the system and the last wolves that are allowed to eat after other wolves finish their meals. The other type of wolf is called delta. They are lower than alpha and beta but are higher than omega. Figure 6 shows the clear hierarchy of the wolves.

Furthermore, wolves are also classified into four main types: sentinels, scouts, hunters, elders, and caretakers. While scouts take responsible for just taking their eyes on the boundaries of the areas and warning whether there are dangers or not, sentinels take responsibility for protecting and maintaining the safety of the pack. Elders, who used to be alpha or beta, are the experienced wolves and will help to give advice on the decision of alpha and beta. Hunters will help the alpha and beta in the job of hunting and finding food. The caretakers take care of the wolves that are weak, and ill.

The hunting steps of the wolves include the following series of actions, which is also the base ideal for the GWO algorithm:

- First, the wolves follow, observe, chase, and get close to the prey.
- Next, they approach, encircle around the prey, and harass it until the prey stops moving around.
- Finally, they attack the stationary prey and finish it.

The first best solution is alpha (*α*), followed by the second and third best solution are beta (*β*) and delta (*δ*), respectively. The rest one is omega (*ω*), who follow the three upper-level wolves’ demands. The GWO algorithm also follows the procedure of hunting prey of grey wolves in real life. Optimization in an algorithm can be considered a hunting action in real life.

As described above, wolves encircle their prey in hunting. In the mathematical model, encircle actions are proposed in the below equations. The wolves’ position is updated by calculating the distance from the prey’s updated position (estimated by *α*, *β* and *δ*) as shown in Equations (2) and (3):

(2) $$\vec{D}=\left|\vec{C}.{\vec{X}}_{p}\left(t\right)-\vec{X}\left(t\right)\right|,$$

(3) $$\vec{X}\left(t+1\right)={\vec{X}}_{p}\left(t\right)-\vec{A}.\vec{D},$$

where *t* is the current iteration, $\vec{X}$ and $\vec{X}$*_p_* is the position vector of grey wolf and prey, respectively. The coefficient vector $\vec{A}$ and $\vec{C}$ are calculated as follows in Equations (4) and (5):

(4) $$\vec{A}=2\vec{a}.\vec{r_{1}}-\vec{a},$$

(5) $$\vec{C}=2.\vec{r_{2}},$$

where $\vec{r_{1}}$ and $\vec{r_{2}}$ is random between 0 and 1. Parameter *a* is linearly decreasing (from 2 to 0), which is measured as equation below:

(6) $$a=2-2\left(\frac{t}{T}\right),$$

where *t* is the number of iterations, and *T* is the maximum of *t*.

**Figure 7:**
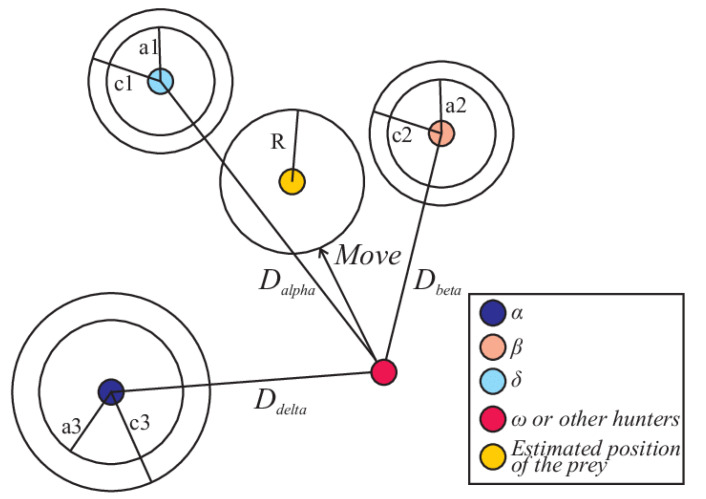
Updated position of a particle in GWO.

As seen in the Figure 7 above, the grey wolf position is adjusted depending on the location of prey. By changing the value of the $\vec{A}$ and $\vec{C}$ vectors, different locations surrounding the optimal agent will be acquired depending on the current position. Hence, to describe the hunting behaviour of grey wolves in a mathematical way, the first three best solutions are saved and applied to the other search agents.

**Figure 8:**
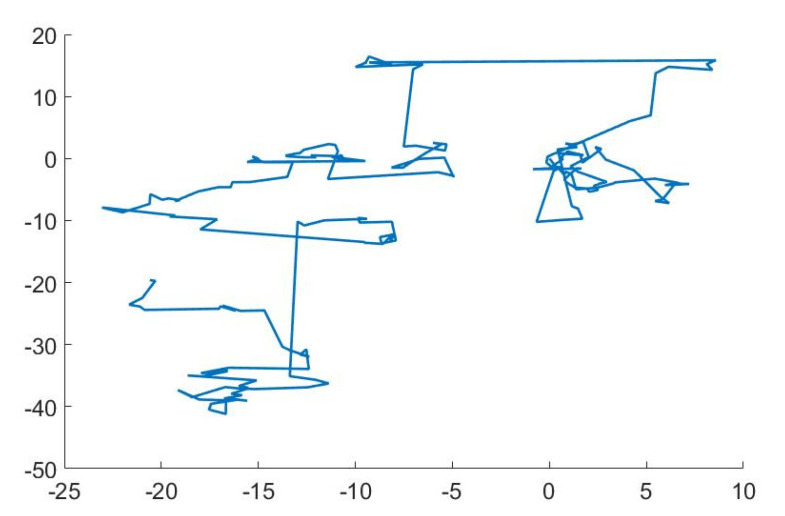
Movement of Levy flights.

Levy flight is a type of non-Gaussian random method in which the step lengths of the random walk have heavy-tailed belongingness. As shown in Equation (7) and Figure 8, the distribution is a simple power-law equation.

(7) $$Levy\left(\beta \right)∼{\left|t\right|}^{-1-\beta };\;0<\beta \;\leq \;2,$$

The span from the random walk’s origin tends to a stable distribution after a certain number of steps. The flight behaviour of many animals and insects reflects the characteristics of Levy flights, according to various surveys and investigations. The rummaging path of a creature is, for the most part, an arbitrary walk because the next move is determined by both the transition probability to the next location and the present condition. The chosen path is heavily reliant on a probability. This enables the formulation of a mathematical model (Kohli et al., 2019).

GWO uses exploration to look for the prey and exploitation to approach its steps to find the entire search space and obtain the optimal solution. This enables the wolf to study the entire search region and find the best solution. Due to an imbalance between the two factors, GWO frequently performs poorly on benchmark functions that are not uni-model. Furthermore, the inadequacy of wolf exploration in some circumstances can lead the model to commit to a local best (optimal). As a result, a new hybrid grey wolf optimization approach has been developed to balance the search space’s variety and intensity.

The Levy flight enhancement aims to incorporate randomness and improve the algorithm’s exploration rate. Individual positions are updated in Levy flight enhanced GWO by integrating the step size as shown in Equation (8).

(8) $$\vec{Y_{t+1}}=\vec{Y_{p}\left(t\right)}+\alpha \;⊗\;Levy\;\left(\beta \right),$$

*α* represents the step size with its value greater than 0, and its changeable scale depends on the problem. In this thesis, α is set to 0.1. The product refers to a multiplication of entries that make the adjustment to the positions. Levy flights take random steps from Levy distribution for big leaps and these are defined as Equations (9)–(11) below.

(9) $$\vec{Y_{t+1}}=\vec{Y_{p}\left(t\right)}+\alpha \;⊗\;Levy\;\left(\beta \right),$$

(10) $$u\;∼\;N\left(0,\sigma_{u}^{2}\right);\;v\;∼\;N\left(0,\sigma_{v}^{2}\right),$$

(11) $$\sigma_{u}=\left\{\frac{\gamma \;\left(1+\beta \right)\;sin\;\left(\Pi \beta /2\right)}{\gamma \left[\left(1+\beta \right)\;/2\right]\;\beta 2^{\left(\beta -1\right)/2}}\right\};\;\sigma_{v}=1,$$

where, *γ*: Gamma function and *β*: control parameter (0.5).

However, for a discrete classification problem, the variables and search space are limited to binary values. Hence, Emary, (2016) [29] said that it is important to attach the wolf position to a binary value. The wolves’ update equation is a product of averaging *Yα*, *Yβ*, *Yδ* position vectors that leads each wolf to the first three optimal. In binary GWO, whether the solution is good or not, it remains in binary form. Hypercube corners constrain all the solutions. This thesis employs a binary enhanced grey wolf optimization (EBGWO) for finding the optimal features. The GWO method’s exploration capabilities is improved by this approach.

(12) $$\vec{X\left(t+1\right)}=\frac{\vec{X_{rand1}}+{\vec{X}}_{rand2}+{\vec{X}}_{rand3}}{3},$$

where *rand* is a uniformly distributed random number, and *X*(*t* + 1) is the updated binary position vector at iteration t. The end values of Levy flights enhanced GWO are continuous; hence, binary conversion from continuous to discrete will be done by employing the sigmoid function below.

(13) X(t+1)={1 if sigmoid (di) ≥ rand 0 otherwise,

(14) $$Sigmoid\left(s\right)=\frac{1}{1+e^{-10\left(x-0.5\right)}},$$

A fitness function is deployed to measure the quality of optimizer solutions and guide the wrapper algorithm. The fitness function should consider both the classification performance and the selected features. The optimal feature subset should yield the highest performance with the smallest amount of selected features. Since the objective is to maximize the model performance and minimize the feature space, the fitness function is used as follows:

(15) $$Fitness=\alpha E_{R}\left(D\right)+\beta \frac{|R|}{|C|},$$

where:

$E_{R}\left(D\right)$ is the classification error rate for the condition attribute set.

*R* is the set of features selected by the classifier.

*C* is the total amount of features.

*α* ∈ [0, 1] and *β* = 1 − *α* are constants to control the importance (trade-off) between the Error rate of the classification performance.

The maximum iteration that Feature Selection with enhanced BGWO will process is 1000 iterations. It is notably that 367 relevant features are selected out of the total original 754 features by using BGWO for feature selection; thus, the feature dimension is reduced by approximately 51%.

## 4. Result Analysis

### 4.1. Performance Metrics

- True positives (TP): the model predicted true, and the actual value is true, meaning the PWP is diagnosed with Parkinson’s disease.
- True negative (TN): the model predicted false, and the actual value is false, meaning the healthy control is predicted to be healthy.
- False positive (FP): the model predicted true, and the actual value is false/TypeI error, meaning the healthy control is inaccurately diagnosed as PWP
- False negative (FN): the model predicted false, and the actual value is false/Type II error, meaning the PWP is inaccurately predicted as healthy control.

Performance metrics including accuracy, precision, recall, *F*1 score, and *AUC* are used to measure the efficacy of these algorithms, as illustrated from Equations (16)–(20) below:

(16) $$Accuracy=\frac{TP+TN}{TP+TN+FP+FN\;},$$

(17) $$Precision=\frac{TP}{TP+FP},$$

(18) $$Recall=\frac{TP}{TP+FN},$$

(19) $$F1=2\frac{precision*recall}{precision+recall},$$

(20) $$AUC=\frac{S_{p}-n_{p}\left(n_{n}+1\right)/2}{n_{p}n_{n}},$$

where:

$S_{p}$: The sum of positive instances ranked;

$n_{p}$: The number of positive instances;

$n_{n}$: The number of negative instances.

### 4.2. Model Results

The Table 2 illustrates the achieved results with slight improvements for the k-NN and SVM algorithm compared to the baseline results and the result from key reference. On the contrary, DT did not perform optimistically, and LightGBM witnessed a decrease in its performance after applying feature selection but still remains the highest accuracy.

KNN and SVM classifiers yielded higher accuracy of 87.8% and 86.6%, respectively. However, k-NN achieved an outstanding Recall score and the fastest processing time. Nevertheless, even with or without the feature selection step, LightGBM still obtains the highest accuracy out of all the algorithms, which is 90.5%, when running the baseline model.

KNN accurately predicted 139 out of the actual 141 cases that got PD, but LightGBM got less total of falsely predicted cases. It is notable that in healthcare examination, false detection of an illness when the patient is not affected by it is considered to have fewer consequences than predicting the sick individual as healthy. Therefore, false alarms are acceptable as long as PWP is not mistaken as healthy. To do so, False Positive, in this case, must be minimized, which leads to a better Prediction score.

**Figure 9:**
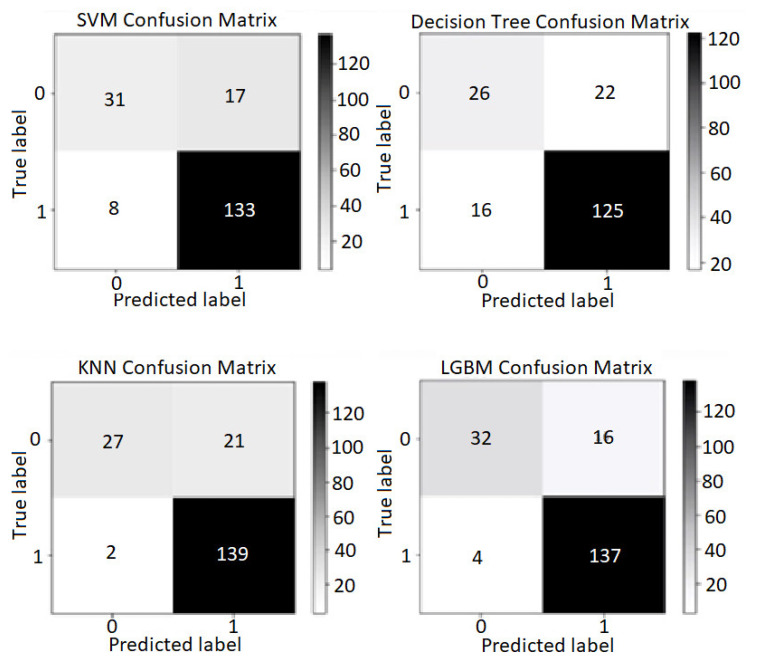
Confusion Matrix of the proposed model.

The results show outstanding Recall score thus allow specialists to detect PD as soon as possible. Meanwhile, Precision scores indicate that correct classification of the patients can lead up to 85–90% of the time. LightGBM obtains the most harmonic balance based on its highest F1 scores between Precision and Recall (93.2% in the proposed model and 93.9% in the Baseline model). The confusion matrix of models is presented in the Figure 9.

The proposed model achieved more promising results than that of the baseline run when it comes to the traditional and most frequently used machine learning algorithms (except for Decision Tree) with reduced dimensions, allowing the process to run quicker. The efficiency of the proposed Gradient Boosting algorithm achieved the best performance with or without feature selection. However, it appears to be best to allow all the features to be trained under the control of LightGBM.

## 5. Conclusions

In conclusion, this thesis has successfully reduced the dimensionality of vocal signal features based on an enhanced metaheuristic model. This leads to an improvement in time processing when using only the important features to feed in the classifiers.

Despite the optimistic results of the proposed model, some limitations must be addressed. This model is only concerned about the symptom of vocal changes and excludes other serious symptoms of PD, making the model lack generalization for PD diagnosis. This can cause the proposed models to poorly generalize and predict. Another side is that even though the model attained good results, it still needs further developments and experiments to be implemented clinically since the patient’s profiles are in much complexity.

Therefore, some recommendations are needed for future works, including solving an over-fitting problem that occurs most of the time when using machine learning models, which is equivalent to solving the generalizability problem mentioned above. An improvement in the accuracy is always needed, especially when considering an imbalanced dataset with high dimensionality. Moreover, instead of dividing the patients into two groups, a multi-classification framework should be developed based on several serious symptoms of Parkinson’s disease. In addition, we also apply SHAP values (Shapley Additive exPlanations), which is a method based on cooperative game theory in order to increase transparency and interpretability of classifiers. We may build SHAP summary plots to show the direction, magnitude, revalence of a feature’s effect and SHAP dependence plots to display the impacts between feature’s value and the prediction output of every samples in this dataset [30].

## Figures and Tables

**Table 1:** Overview of the feature sets.

Title 1	Measure	Explanation	# of Feature
Baseline features	Jitter variants	Capture instabilities of the oscillating pattern of the vocal folds & its subset quantify the cycle-to-cycle changes in the fundamental frequency.	5
	Shimmer variants	Capture instabilities of the oscillating pattern of the vocal folds & its subset quantify the cycle-to-cycle changes in the fundamental amplitude.	6
	Fundamental frequency parameters	the frequency of focal folds vibration. Mean, median, standard deviation, minimum & maximum values were used	5
	Harmonicity parameters	Due to incomplete vocal fold closure, increased noise components occur in speech pathologies. Harmonics to Noise Ratio and Noise to Harmonics Ratio parameters quantify the ratio of signal information over noise, which were used as features.	2
	Recurrence Period density Entropy (RPDE) I	Provides information about the ability of the vocal fold oscillations and quantifies the deviation form F0	1
	Detrended Fluctuation Analysis (DFA)	Quantifies the stochastic self-similarity of the turbulent noise.	1
	Pitch Period Entropy (PPE)	Measures the impaired control of fundamental frequency F0 by using a logarithmic scale	1
Time-frequency features	Intensity Parameters	Related to the power of speech signal in dB. Mean, minimum and maximum intensity values were used	3
	Formant Frequencies	Amplified by the vocal tract, the first four formants were used as features.	4
	Bandwidth	The frequency range between the formant frequencies. The first four bandwidths were used as features.	4
Mel frequency cepstral coefficients (MFCCs)	MFCCs	Catch the PD effects in the vocal tract separately from the vocal folds	84
Wavelet Transform based Features	Wavelet Transform (WT) features related to F0	Quantify the deviation in F0	182
Vocal fold features	Glottis Quotient (GQ)	a measure of periodicity in glottis movements that provides information about the opening and closing duration of the glottis	3
	Glottal to Noise Excitation (GNE)	Quantifies the extent of turbulent noise caused by incomplete vocal fold closure in the speech signal.	6
	Vocal Fold Excitation Ratio (VEER)	Quantities the amount of noise, produced due to the pathological vocal fold vibration using non-linear energy at entropy concepts.	7
	Empirical Mode Decomposition (EMD)	Decompose a speech signal into elementary signal components by using adaptive basis functions &	
	Energy/entropy values obtained from these components are used to quantify noise.	6	
Tunable Q-factor Wavelet Transform (TQWT)	TQWT	A more extensive quantification method for fundamental frequency deviation as compared to WT I	432

**Table 2:** Results comparison between baseline model and proposed model.

Classifier		Accuracy	Precision	Recall	F1-Score	AUC	Computational Time
k-NN	Baseline model	0.862	0.857	0.979	0.914	0.75	0.443
	Proposed model	0.878	0.869	0.986	0.923	0.316	0.878
SVM	Baseline model	0.841	0.840	0.972	0.901	0.71	0.560
	Proposed model	0.866	0.887	0.943	0.914	0.527	0.866
DT	Baseline model	0.810	0.878	0.865	0.871	0.76	0.640
	Proposed model	0.795	0.850	0.886	0.868	0.744	0.795
Proposed LGBM	Baseline model	0.905	0.902	0.979	0.939	0.83	2.056
	Proposed model	0.894	0.895	0.972	0.932	1.926	0.894

## Data Availability

The dataset used were anonymous data obtained from https://archive.ics.uci.edu/ml/datasets/Parkinson%2BSpeech%2BDataset%2Bwith%2B%2BMultiple%2BTypes%2Bof%2BSound%2BRecordings (Last accessed on 15 June 2022).
